# Possibilities for the peer review of interdisciplinary research: a small-scale qualitative study

**DOI:** 10.1186/s41073-026-00204-3

**Published:** 2026-06-16

**Authors:** Georgia Vesma

**Affiliations:** The University of Manchester, Manchester, England

**Keywords:** interdisciplinarity, anonymous peer review, open peer review

## Abstract

**Background:**

Despite critiques from across academia, double-anonymised peer review is still considered the ‘gold standard’ quality-assurance tool for the publication of academic research. While some journals make use of ‘open’ peer-review practices to make elements of academic publishing more transparent, anonymised is still used by the majority of academic journals, with anonymity considered the best guarantor of reviewer objectivity. Anonymised peer review remains a ‘black box’ which conceals diverse and idiosyncratic practices by reviewers, editors and journals. This paper seeks to uncover the impact of such practices on the peer review of interdisciplinary research.

**Methods:**

The author first conducted a survey of 164 self-identified interdisciplinary researchers from a range of disciplines at one research-intensive UK university to identify barriers to and enablers of high-quality peer review for interdisciplinary papers. This quantitative/qualitative survey was supplemented by interviews with authors, editors and reviewers from across biological and medical sciences, physical sciences and engineering and social sciences and humanities.

**Results:**

Authors of interdisciplinary research papers reported experiencing disadvantage in peer review practices, including perceived bias against interdisciplinary research by mono-disciplinary reviewers and the assignment of unsuitable reviewers. Reviewers reported being assigned papers to review that were not within their field of expertise, and insufficient editorial guidance on review processes for interdisciplinary papers. Editors reported that the ‘reviewer crisis’ has further complicated the already challenging process of seeking peer review for interdisciplinary research.

In interviews, reviewers shared strategies to overcome weaknesses in the review process for interdisciplinary papers. These included confidence reporting where the reviewer did not feel able to comment on all aspects of the paper, and seeking advice from colleagues to enhance their review. Practices were employed with varying degrees of formality, and in some cases undermined the stated goals of anonymised peer review.

**Conclusions:**

Considering the possible implications of the informal strategies employed by peer reviewers, the paper proposes a framework for interdisciplinary peer review informed by existing open peer review practices that formalises and incorporates existing strategies used by reviewers and editors to enhance the peer review of interdisciplinary research papers.

## Introduction

Interdisciplinary research, far from being an outlier or rarity in academic research, has been recognised for decades as a possible route to solving global problems [1, 2]. Despite this, there remains an impression that interdisciplinary research lacks coherent frameworks for assuring quality [1, 3, 4]. It follows that the outputs of interdisciplinary research may face additional barriers to publication in peer-reviewed journals when compared to mono-disciplinary publications.

Quality in published research outputs is supposedly ensured by the framework of anonymised peer review. Differing degrees of anonymisation are employed across fields and individual journals. ‘Single anonymisation’, where author identities are known to reviewers but not vice-versa is the most common [5]; ‘double anonymisation’ where author and reviewer identities are obscured for all parties except editors is most popular in the social sciences and humanities; and ‘triple anonymisation’ [5, 6], where editors are also not apprised of author and reviewer identities is employed by a small number of journals. Double anonymisation, still considered the ‘gold standard’ quality assurance mechanism [7] has been subject to a wide variety of critiques, not least of which is the assertion that double-anonymised peer review does not seem to significantly affect the rate of errors occurring in published research [8].

While the ubiquity of anonymised peer review (whether single or double anonymised) might suggest a monolithic, consistent practice, there is significant evidence that the ‘black box’ [9] conceals a diverse and idiosyncratic set of practices on the part of reviewers and editors. Since the 1990 s, ‘open’ peer review has been proposed as a route to increase transparency and incentivise good practice within the reviewing process. Open peer review, however, does not describe one process but rather a constellation of overlapping practices which are applied differently by different journals [10]. Indeed these practices can be understood as part of a larger spectrum of peer review practices, with ‘triple anonymised’ review at one end and ‘open participation’ [10] peer review at the other in terms of transparency. These differing approaches all offer different benefits and advantages, making them more or less suited to different types of research publication. This study has focused on the ways in which double- and single- anonymised peer review are perceived to affect outcomes and experiences for interdisciplinary research publications.

Many of the critiques levelled against anonymised peer review are equally applicable for mono- and multi-disciplinary publications. Interdisciplinary research nevertheless poses specific challenges for peer review, particularly around selection of appropriate reviewers and the integration of reviewer perspectives into productive feedback. There has thus far been only limited enquiry into how traditional peer-review practices for scholarly applications might interact with a growing body of interdisciplinary research [11, 12] and systematic reviews of open peer review practices have not ventured outside of disciplinary categories [10, 13, 14].

### Literature review

Both interdisciplinary research and peer review have been the subject of scholarly interrogation for multiple decades, with communities of study developing alongside the practices themselves. In addition to theoretical interrogations of interdisciplinary research [15, 16], a growing body of research into interdisciplinary research practices has flourished alongside the practices themselves as interdisciplinary scholars have turned their attention to their own ways of working [17–19]. These investigations focus on *research* practices: ways of communicating and collaborating across disciplinary divides, building relationships of trust and understanding in order to gather, analyse and integrate data. Where publication is addressed, the focus is often on language [17] rather than structural challenges for the publication of interdisciplinary research – beyond which journals to target [20].

Research into the challenges of *publishing* inter- (and trans- and multi-) disciplinary research has often narrowly focused on questions of authorship. Authorship and crediting practices differ from discipline to discipline and from journal to journal [21]. Author order and inclusion, already a contentious question [22, 23] is made yet more contentious at the interface of disciplines with different norms. This focus on author credit – “a determining feature of career success” [21] – bypasses the peer-review process as a key stage in the life cycle of interdisciplinary research. This in turn contributes to an existing ‘survivor bias’ issue: unpublished or rejected articles are absent from the data. While this problem is not unique to the study of interdisciplinary publications [24], it makes it difficult to understand whether interdisciplinary research suffers from quality problems or is affected by bias from reviewers sceptical of interdisciplinary approaches.

In 2020 Jonathan Tennant & Tony Ross-Hellauer characterised the emerging field of peer review studies as ‘critical and interdisciplinary’ [7]. Their field-shaping article identified key areas for further interrogation including the notions of ‘expertise’ and the impact of epistemic diversity (or lack thereof) between reviewers. Despite these topics being highly relevant to the peer review of interdisciplinary research, Tennant & Ross-Hellauer do not make explicit reference to publications with authors from multiple disciplines when discussing ‘the limitations to our understanding of peer review’. While subsequent studies have taken up their call for deeper interrogation of peer review practices [14, 25, 26] the challenges of peer review for interdisciplinary research remain to some extent unknown.

Research on the peer review of interdisciplinary research largely focuses on practices of research grant review [2, 27–29] rather than publication review [11]. This focus is born in part out of the requirements for greater transparency in grant review, which is typically a panel-based process, and often requires panels to fully justify their decisions. Data is therefore more accessible, facilitating interrogation of the processes. What Smith [9] termed the ‘black box’ of anonymised peer review for academic publication makes meaningful, systematic investigations into review practices and editorial decisions extremely challenging. Lee Sigelman’s approach, using data from his role as editor at the *American Political Science Review* to assess the impact of multiple authorship on success rates for papers submitted to the journal, addresses one of the major challenges of interrogating interdisciplinary research through publication and citation statistics: “…citation studies are confined to published papers, leaving open the question of whether multi-author papers are more likely to pass the prior hurdle of withstanding a rigorous review process and being deemed worthy of publication in the first place” [12]. Sigelman found that multiple-discipline authorship had a slightly positive effect on a paper’s chances of publication – but this case is limited to one journal in the social sciences.

Scholarship on open peer review practices for publications, on the other hand, does not engage deeply with interdisciplinarity. This is illustrated by Ross-Hellauer’s thorough 2017 cataloguing of the ‘types’ of open peer review (including ‘open identities’, ‘open reports’ and ‘open participation’) described in academic publications. Ross-Hellauer is attentive to discipline to some degree, noting distinctions between ‘STEM’ (Science, Technology, Engineering and Medicine) and ‘SSH’ (Social Science and Humanities) practices of open peer review. His tables and graphs indicate that 37.7% of the definitions of open peer-review he encountered were ‘interdisciplinary’ – used here to mean either discipline-agnostic, or spanning both ‘STEM’ and ‘SSH’. It should be noted that this definition of ‘interdisciplinarity’ excludes disciplinary collaborations *within* those broad acronyms, which describes a great deal of published interdisciplinary research. Ross-Hellauer’s subsequent categorisation of definitions of ‘open peer review’ uses the ‘STEM’ and ‘SSH’ categories to inform the analysis; despite the large number of ‘interdisciplinary’ sources drawn upon, interdisciplinary research does not feature in the conclusions. Fitzpatrick and Santo’s 2012 report [13] for the Andrew W. Mellon Foundation focuses solely on the humanities disciplines, although the authors do note that an adoption of open peer review practices would open up opportunities for greater interdisciplinarity when compared to traditional anonymised peer review. Karhulahti and Backe’s 2021 qualitative study [14] with editors in the social sciences and humanities explores resistance to open peer review in these disciplines, noting that discipline-specific matters have implications for peer review practices. The uneven uptake of open peer review across disciplines is one example of how disciplinary frameworks can have implications for the publication of interdisciplinary research.

This article therefore aims to bring the study of peer-review practices and the study of interdisciplinarity into closer conversation and nuance existing discussions about anonymised peer review and open peer review by incorporating interdisciplinary perspectives. Employing qualitative data gathered from reviewers, authors and editors in parallel, it will take a multi-perspective approach to the ‘problems’ identified. Considering the possible implications of idiosyncratic review practices for the fair review of interdisciplinary research, this article will put forward a possible framework for interdisciplinary peer review informed by existing open peer review practices.

Offering an early first step in addressing this gap, this article draws on a survey and interviews conducted within one research-intensive UK university to understand how authors, reviewers and editors experience and approach the review of explicitly interdisciplinary publications. It examines emerging themes: lack of (inter)disciplinary expertise among reviewers, inconsistent editorial practices, and a lack of training and support for reviewers. While recognising that these challenges also affect the assessment of mono-disciplinary publications, this article calls for deeper consideration of how interdisciplinary publications are assessed, proposing that ‘interdisciplinary peer review’ might be considered an interdisciplinary practice in itself.

While definitions of ‘interdisciplinarity’ abound [30, 31], for the purposes of this article (and the survey from which it draws data) ‘interdisciplinary research’ is very broadly defined as research that involves two or more academic disciplines (this was phrased in the survey as ‘research and publication which crosses disciplinary boundaries’). An ‘interdisciplinary publication’ is defined as a written output intended for publication for a scholarly audience, with more than one author and with more than one discipline represented in the authorial team. These definitions are simplistic and do not account for the great diversity of opinion on what is ‘interdisciplinary’ (as opposed to multi- or trans-disciplinary) research; they are intended instead to capture a broader range of activity from a small dataset.

To understand how the anonymised peer review process impacts on interdisciplinary publications, this paper will combine data from authors with data gathered from reviewers and editors. By looking at the process from multiple perspectives, it will aim to define the major challenges of anonymised peer review for interdisciplinary research and propose possible solutions informed by the realities of academic peer-review.

## Methods

The methods employed for this research should be contextualised with a brief note about the author and researcher. The dataset for this paper was gathered as part of an Open Research Fellowship alongside my full-time Professional Services role at a large, research-intensive UK University. My work in Research Development focuses on securing research funding, based largely within Social Sciences and Humanities but with a significant element of interdisciplinary facilitation. This role has informed my approach to this research and, as discussed below, also shaped my data gathering.

In order to create a base for my qualitative research, I first circulated by email a survey across all departments of the University, accompanied by text that invited ‘interdisciplinary researchers’ of all career stages to complete the survey. The survey used the Qualtrics survey platform to gather, store and analyse responses. The broad definition of interdisciplinarity outlined in the background section of this article was employed: the respondent welcome page stated that “The purpose of this research is to explore the barriers and enablers of research and publication that crosses disciplinary boundaries. Although the term ‘interdisciplinary’ is used throughout this survey, this may also include trans-disciplinary or multi-disciplinary research.” The survey employed quantitative questions, such as prompting respondents to score experiences of peer review out of 10, alongside qualitative free-text response fields where respondents could indicate what they thought made for a good (or bad) peer review experience for interdisciplinary publication.

This resulted in survey completion by 164 self-identified interdisciplinary researchers from PhD students to established professors, representing a broad sample of the University’s academic staff. As all participants identified themselves as interdisciplinary scholars, the location of their substantive research role within the structure of the University is here used as a proxy for their disciplinary ‘base’ – but it must be noted that this is an imperfect measure that fails to account for cases such as a computer scientist employed on a Digital Humanities project, or a trained engineer working within a biomedical department (Tables 1 and 2).

**Table 1:** Survey respondents by self-identified ‘career stage’ and disciplinary base

			Self-identified ‘career stage’			
		**Respondents**	**PhD**	**Early-Career**	**Mid-career**	**Established**
	**Total**	164	9	41	38	60
Substantive research role based in:	Biological and medical sciences	32	3	10	4	11
	Physical sciences & engineering	21	1	5	8	5
	Social sciences & humanities	111	5	26	26	44

**Table 2:** Peer-review role experience of survey respondents (note individuals often held multiple roles)

	Reviewee	Reviewer	Editor
Total	82	61	28
Biological and medical sciences	19	10	2
Physical sciences & engineering	9	8	3
Social sciences & humanities	54	43	23

Survey participants were able to self-select for participation in semi-structured follow-up interviews. These were conducted with 12 participants in total: 3 whose substantive research role fell within biological and medical sciences, 3 within physical sciences and engineering, and 6 within social sciences and humanities. Here again my position as a (distant, junior) colleague of the surveyed population likely impacted on the degree of engagement. More than 50% of interviewees were people I had previously worked with in some capacity. All 12 interviewees had acted as authors on interdisciplinary publications; 10 had acted as reviewers; 4 had acted as editors. Across all disciplines these roles are mobile with an expected trajectory (from author to reviewer to editor) across the career of the individual. Interviewees who held multiple roles were encouraged to consider how this experience shaped their decision-making. Transcripts of these interviews have been drawn upon for this paper, supplemented by quantitative observations drawn from the survey as well as analysis of free-text survey responses.

## Results

### Who is contributing to interdisciplinary research publications, and how are they experiencing anonymised peer review?

The survey asked respondents to identify if they had been a ‘named contributor to an interdisciplinary article that underwent peer review’. The term ‘named contributor’ was chosen as the most inclusive way to capture participation in interdisciplinary research publication – encompassing multiple roles as outlined in the CRediT Taxonomy [32], which was linked in the survey question to ensure respondents understood what constituted a ‘contributor’ (Table 3).

**Table 3:** Participation as a ‘named contributor’ on any interdisciplinary research publication

		**Self-identified as having contributed to a peer-reviewed IDR article**	
**Respondent Group**	**Number of respondents**	No	%
Total	164	82	50%
**Disciplinary ‘base’**			
Biological and medical sciences	32	19	59%
Physical sciences & engineering	21	9	43%
Social sciences & humanities	111	54	48%
**Self-identified career stage**			
PhD	9	5	56%
Early-career	41	20	49%
Mid-career	38	20	53%
Established	60	37	62%

Only 50% of the surveyed self-identified ‘interdisciplinary researchers’ had actively contributed to interdisciplinary research publications. This may be attributable to negative perceptions of interdisciplinary co-authoring and publication – for example, having interdisciplinary publications is not seen as having a positive effect on academic careers [31]. Small sample size notwithstanding, self-identified interdisciplinary academics in the biomedical sciences reported contributing to peer-reviewed interdisciplinary research more commonly than their counterparts in physical sciences and engineering and social sciences and humanities, possibly reflecting the broader culture of publishing in these areas.

‘Early career’ researchers, post-PhD but in the foundational stages of their career, were the least likely to have contributed to an interdisciplinary research publication – less so even than PhD researchers. This may indicate that this cohort prioritised the career security offered by publishing in their disciplinary ‘base’, which is linked to stronger promotion cases [31, 33]. Conversely ‘established’ researchers, the largest career stage cohort, were the most likely to have contributed to interdisciplinary research publications. This may be a produce of a number (or combination) of factors including longer and generally more diverse publication histories; reduced concern about promotions criteria; and established researchers receiving more invitations to collaborate from those keen to access their domain expertise or reputation. Across all disciplinary bases and career stages, among this limited sample, it can be said that about half of researchers who consider themselves to be engaged in interdisciplinary research are also contributing to interdisciplinary publications.

A question format with multiple selections permitted respondents to identify whether their co-authored article(s) were ultimately accepted for publication (Table 4):

**Table 4:** Contribution to/outcome of interdisciplinary research publications by disciplinary ‘base’ and self-identified career stage

I have been a named contributor to an interdisciplinary article that underwent peer review, and was ultimately	Total	accepted for publication	not accepted for publication	Ticked both boxes (multiple articles)
Total	82	42 (51%)	2 (2%)	38 (46%)
Disciplinary ‘base’				
Biological and medical sciences	19	10 (53%)	1 (5%)	8 (42%)
Physical sciences & engineering	9	6 (67%)	0	3 (33%)
Social sciences & humanities	54	26 (48%)	1 (2%)	27 (50%)
Self-identified career stage				
PhD	5	4 (80%)	0	1 (20%)
Early-career	20	11 (55%)	1 (5%)	8 (40%)
Mid-career	20	9 (45%)	0	11 (55%)
Established	37	18 (49%)	1 (2%)	18 (49%)

The majority of respondents who had contributed to a peer-reviewed interdisciplinary research article had at least one such publication accepted by a journal. Career stage correlated, as might be expected, to more mixed outcomes – 50% of mid-career and established scholars had experienced both acceptances and rejections, which can be seen as the product of having made more attempts.

Respondents who had experience of peer-review on an interdisciplinary research publication were subsequently given the option to score their experience as a reviewee with an integer on a scale from −5 (labelled ‘entirely negative’) to + 5 (‘entirely positive’). Respondents who did not complete this question have been omitted from the table (Table 5).

**Table 5:** Breakdown of responses to question “how would you characterise your experience of interdisciplinary peer review as a reviewee?”

Respondent Group	No	Low	High	Mean	Mode	Median
Total	77	−4	5	1.7	3	3
**Disciplinary ‘base’**						
Biological and medical sciences	16	−4	5	1.3	N/A	1
Physical sciences & engineering	9	−3	4	1.5	N/A	2
Social sciences & humanities	52	−2	5	1.9	3	3
**Self-identified career stage**						
PhD	4	−4	4	0	0	0
Early-career	18	−1	4	1.6	0	2
Mid-career	19	−2	5	1.8	3	3
Established	36	−3	5	1.9	3	3
**Experience of rejection after peer-review**						
No rejection after PR reported	42	−4	5	2.4	3	3
At least one rejection after PR reported	35	−3	5	1	0	0

The modal score across all respondents was 3 (‘mostly positive’), suggesting that most contributors felt good about their experience of anonymised peer review. The mean score of 1.7 (between ‘neutral’ and ‘mostly positive’) being lower than the modal score suggests that a negative experience of peer review had a strong effect on a small but relevant group.

Breaking the scores down by disciplinary ‘base’ reveals that the disproportionate engagement of social science and humanities researchers has likely shaped this result – this cohort was happier with their experience of anonymised peer review than their counterparts in other fields, supporting Karhulahti and Backe’s 2021 findings [14].

Career stage of respondent showed some correlation with a more positive experience of the peer-review process for interdisciplinary research. Despite good success rates for papers co-authored by PhD researchers, their overall experience of the process was ‘neutral’. Mid-career and established researchers, though more likely to have received rejections overall, had more positive impressions of the peer-review process.

As might be expected, experience of a rejection did have an effect on scores, significantly driving down the modal experience score to 0 (‘neutral’) although the most negative experience in this respondent group was not rated as low as the most negative experience within the no-rejections group.

### Who is reviewing interdisciplinary research publications, and how are they experiencing anonymised peer review?

Survey respondents were asked to identify if they had “acted as a peer reviewer for a journal, and… reviewed article(s) with named contributors from multiple disciplines” and/or if they had “held an editorial role for a journal, and… assigned articles with named contributors from multiple disciplines for peer review.” Responses were as follows (Table 6):

**Table 6:** Participation as peer-reviewer/editor on any interdisciplinary research publication

		**Have peer-reviewed IDR paper(s)**		**Have assigned peer reviewers for IDR paper(s)**	
**Respondent Group**	**Number of respondents**	**No**	**%**	**No**	**%**
Total	164	61	37%	28	17%
**Disciplinary “Base”**					
Biological and medical sciences	32	10	28%	2	6%
Physical sciences & engineering	21	8	38%	3	14%
Social sciences & humanities	111	43	39%	23	21%
**Career Stage**					
PhD	9	2	22%	0	0%
Early-career	41	9	22%	3	7%
Mid-career	38	14	37%	5	13%
Established	60	34	57%	20	33%

Bearing in mind the small sample size and skew toward more established academics and social sciences and humanities, these figures tell a coherent story. All respondents who had held reviewer or editorial roles also reported having had experience as authors of interdisciplinary research papers. Experience as reviewer or editor scales with career stage, and all respondents who had held editorial roles also had experience as a reviewer of interdisciplinary research. Social science and humanities academics reported being between 1.5 and 3 times more likely to have assigned interdisciplinary research papers for review than their counterparts in other disciplinary bases. Speculatively, this may be due to journals with a social science leaning receiving a large number of submissions from multi-disciplinary author teams, or having more ‘open’ desk-assessment criteria that allow more papers to make it to anonymised peer review.

Reviewers with experience on at least one interdisciplinary research publication were given the option to score this experience with an integer on a scale from −5 (labelled ‘entirely negative’) to + 5 (‘entirely positive’). Respondents who did not complete this question have been omitted from the table (Table 7).

**Table 7:** Breakdown of responses to question “how would you characterise your experience of interdisciplinary peer review as a reviewer?”

Respondent Group	No	Low	High	Mean	Mode	Median
**Total**	57	0	5	2.4	3	3
**Disciplinary ‘base’**						
Biological and medical sciences	9	0	5	2.8	3	3
Physical sciences & engineering	8	0	4	1.6	0	1.5
Social sciences & humanities	40	0	5	2.6	3	3
**Self-identified career stage**						
**PhD**	2	0	3	1.5	N/A	N/A
**Early-career**	8	0	4	2.5	4	3
**Mid-career**	13	0	4	2.1	3	3
**Established**	33	0	5	2.6	3	3

With no score below 0 (‘neutral’), it can be inferred that the reviewers who responded to the survey did not find the experience of reviewing interdisciplinary research papers to be negative. Almost across the suite of responses the modal and median experience score was 3 (‘mostly positive’) with reviewers in physical sciences and engineering tending more toward a neutral opinion of their experiences.

To understand what underpins these quantitative responses, respondents from all groups (authors, reviewers and editors) were given the option to give up to three free-text responses each to the questions “What factors do you think might support high-quality peer review for interdisciplinary publications?” and “What factors do you think might be a barrier to high-quality peer review for interdisciplinary publications?” These two questions garnered 271 responses in total, and these were sorted into broad categories (Table 8):

**Table 8:** Overview of responses to free-text questions on enablers of/barriers to high-quality peer review of interdisciplinary publications, with example answers – enablers first, barriers second

Heading	Number of responses	Example responses
Reviewer behaviour/characteristics	86	“openmindedness” “reviewers unable to look past their own discipline”
Reviewer selection by editors	70	“Reviewers with varied backgrounds/from different disciplines” “Reviewers that cannot adequately cover the disciplines and interdisciplinary material involved in the paper”
Editorial support	26	“acknowledgement of the value of inter-disciplinary research in relevant journals' aims” “Editorial "gatekeepers" who don't value interdisciplinary work”
Provision of guidance for reviewers	23	“Detailed and accessible guidance for reviewers (this would be helpful for authors too!)” “lack of guidance for reviewers”
Open review practices	13	“Transparency (reviewers should be named)” “Lack of communication between reviewers”
Journal selection by authors	10	“Finding the right journal” “Mono-disciplinary journal”
General matters affecting interdisciplinary research or reviewing practices	43	“More focus on impact for policy and practice” “Not enough word count to do needed explanations justice”
TOTAL	271	

There was no major distinction between the types of issues respondents identified as ‘enablers’ or ‘barriers’: ‘barriers’ were often expressed as the inverse of a stated ‘enabler’. It can be surmised that the most important factors affecting high-quality peer review for interdisciplinary research are related to *reviewer behaviour* and *reviewer selection by editors.*

Responses coded as *reviewer behaviour* describe the attitudes, behaviours and traits of individual reviewers, including the figure of ‘Reviewer 2’, who represents unfair, disengaged and antagonistic review practices [34]. The actions of individual reviewers are perceived to have a significant impact on the process, with a single reviewer able to have a strong negative effect on publication outcomes. The most commonly cited positive trait for reviewers was being open to ideas beyond their home discipline (termed, variously, “open mindedness”, “flexible and open thinking”, “willingness to accept boundary crossing work”). Accordingly, negative reviewer influence was attributed to “Limited desire to understand approaches/issues beyond one's own discipline”, “Failure to take seriously the norms and assumptions of other disciplines”, “disciplinary conservatism” and “just not ‘getting it’ [interdisciplinary research]”. It seems that even one reviewer who is hostile to, or even simply inexperienced with, interdisciplinary research practices is enough to have a negative impact on the anonymised peer review experiences of interdisciplinary research contributors.

The solution to issues caused by reviewer behaviour, according to survey respondents, lies in the reviewer selection process and the provision of guidance for reviewers. When examining these responses it is worth recalling that many survey respondents have experience on both sides of the review process, and several also reported holding editorial positions. This experience likely influenced responses, providing insight into how *reviewee experience* influences *reviewer practices* and ultimately *editorial practices*.

### Reviewer selection for interdisciplinary research

Reviewer selection was mentioned in 26% of survey responses. Some focused simply on the number of reviewers (“enough reviewers”; “need more reviewers than usual”), perhaps to create a wider disciplinary spread or to mitigate against the influence of a single hostile reviewer. There was recognition that the ‘reviewer crisis’ [35] makes assembling an adequately experienced reviewer panel challenging: “A large enough interdisciplinary community such that those [qualified] referees can be sought in the first instance” and “Having enough knowledgeable reviewers available” were both cited as prerequisites for high-quality peer review of interdisciplinary research. While the shortage of reviewers is an issue that affects all peer-reviewed publications, the challenge of assembling a review panel is compounded for editors handling interdisciplinary papers, who are unlikely to have access to a wide enough network of expertise across multiple domains.

Many comments focused on the diversity of the reviewer panel in terms of expertise and knowledge. High-quality peer review for interdisciplinary research is perceived to require “diverse disciplinary expertise”. Some felt that reviewers of interdisciplinary research should themselves be interdisciplinary scholars (“reviewers with… an understanding of interdisciplinary methods”; “Reviewers who have themselves conducted and published interdisciplinary research”; “DEMONSTRATABLE *(sic)*… TRACK-RECORD OF INTERDISC.”) The assumption is perhaps that, having experienced the challenges of interdisciplinary research themselves, reviewers would be more sympathetic and *open* to interdisciplinary research papers, even if reviewing for a disciplinary journal.

Other responses focused on assembling expertise from multiple disciplines into one team: “good choice of reviewers from more than one relevant discipline”; “interdisciplinary review team as well.” Seeking ‘coverage’ of the disciplines represented in the paper was a common suggestion, with respondents noting “Availability of separate reviewers who collectively cover the particular disciplines in question” and “Reviewers with some knowledge or interest in all/most areas covered by the paper” as enabling factors. A small number of comments coded to other themes add nuance to these statements. Even a suitably diverse review team may not produce good results if “lack of communication between reviewers” (coded as *Open review practices*) leads to “Inconsistent feedback between reviewers on the same paper” (coded as *Editorial support,* reflecting the role of editors in mediating reviews.)

The interview data gathered for this project corroborates the survey findings. Authors, reviewers and editors all highlighted reviewer expertise as a significant factor affecting the quality of peer review for interdisciplinary research. A reviewer working at the interface of engineering and the biomedical sciences summed up the challenge:

“…because the papers are interdisciplinary… reviewers are unlikely to be able to review all the whole paper. (…) some of the journals… there's an argument to say that they're not very good journals because the editors are not… finding the appropriate peer reviewers.”

*Established physical science reviewer interview*

This situation has been recognised for almost two decades. As Laudel [29] puts it, “since interdisciplinary research is a new synthesis of expertise, peers in the strong sense of the word do not exist”. Assembling a ‘peer’ review panel for a paper that successfully integrates multiple disciplines in a novel way poses significant challenges even before any question of the ‘reviewer crisis’ is raised.

For their part, editors highlighted the challenges of securing *any* reviewers in the context of paper proliferation and reduced reviewer availability. One editor from the social sciences noted in interview that:

“…some are lucky, some you only have to give four or five invites to get a couple of reviewers. More typically it’s fifteen or twenty. In some instances, you're looking at eighty, ninety, a hundred invitations... So the idea that you particularly manufacture that around a very fine-grained understanding of which forms of disciplinary expertise? No. It doesn't happen.”

*Established social science editor interview*

The difficulty of assembling a suitably qualified interdisciplinary reviewer team is behind one practice that takes place within the so-called ‘black box’. Reviewers from a variety of discipline bases and career stages described asking colleagues from other disciplines for advice and help with peer-reviewing interdisciplinary research. Senior researchers with access to networks were particularly well-placed to do so:

“…the group that I lead… includes physicians… psychologists, statisticians, people who have an engineering background, people who have… AI and technology backgrounds. So what I tend to do, if I really struggle, I either discuss it with colleagues or… even ask them to see whether they can have a look at the paper… I'm not sure whether it's officially allowed. But I do at times ask for… opinions from others.”

*Established biological sciences reviewer interview*

This approach is particularly informal, and the admission that the reviewer is “not sure whether it’s officially allowed” indicates that they are not notifying journal editors when they engage in this practice. This has implications for the practice of anonymised peer review, and goes against ethical best practice [36]. Even overseeing editors may not have the complete picture on who is actually conducting the work of reviewing; that work, already often uncredited, may be taking place through unrecorded channels. This not only undermines confidence in the process, it may expose unpublished research to the risk of untraceable academic malpractice or plagiarism.

A mid-career academic working in the physical sciences recounted a specific example of seeking help with a review, and trying to go through formal channels to do so. This reviewer works in a discipline where single-reviewer peer review is the norm, which presents challenges for the review of interdisciplinary research. To address this, the reviewer tried to ensure colleagues’ feedback was incorporated formally:

“…what I did was I asked some colleagues… and put their replies into my referee report and then sent that back to the journal and said, “look, I think you should probably consult one of these other people to get a second opinion on this. I've done as far as I can.”

And I think actually… the editor didn't do that. He just took the snippets of their advice that I'd sent him and passed it on to the authors. And the authors responded to that. In the end, it did get published. I was a little bit reluctant to publish it.”

*Early-career physical sciences reviewer interview*

When their suggested course of action was not followed, the reviewer reports feeling doubtful that the final article was fit for publication. This anecdote raises further questions about the suitability of double-anonymised peer review for the review of interdisciplinary research. If editors are unwilling or unable to seek additional opinions where reviewer confidence is low, the quality and relevance of reviews available to authors of interdisciplinary research papers may be lower than for single-discipline papers.

These narratives of *informal collaborative review* hint at one possible route for the peer review of interdisciplinary research. Reviewers interviewed for this project did not report being able to communicate with their fellow reviewers while reviewing interdisciplinary research. This is consistent with double-anonymised peer review practices and many forms of open peer review. Where reviewers had the opportunity to see reports from other reviewers, this was reported fairly positively. One early-career researcher currently working in the biomedical sciences noted that “if it is possible to see the other [reviewers’] comments, it doesn't… detract from anything. It may actually help [confirm] you're not just going on a tangent.” This approach to ‘validating’ concerns may be particularly helpful for less experienced reviewers, although without dialogue and mediation it may also result in convergent reviews or cause reviewers to doubt what may be valid critiques.

Reviewers also reported using *confidence reporting* as a strategy where they were invited to review papers outside their expertise. Perhaps due to their experiences as authors of interdisciplinary research papers, reviewers were conscious that a reviewer without relevant expertise could influence outcomes in unfair ways. As a mid-career physical science reviewer remarked, “…a little bit of knowledge is a dangerous thing… it's more important in interdisciplinary work to demarcate your own expertise” (emphasis mine). For some reviewers, confidence reporting also represented a way to try and influence the makeup of the review team:

“…a lot of the journals… have a box where they would ask you “what aspects of this are you confident about and what aspects of this are you less confident about?”… or there is a box where you can add notes. So you could say, “well, I don't know about this, this, this”… the overall review process will cover all of the aspects and I personally think it's the duty of editors… to make sure they get a broad set of reviewers.”

*Early-career biological sciences reviewer interview*

It should be noted that while this reviewer found that “a lot of” the journals they reviewed for have a box for this purpose, a reviewer at the interface of social and physical sciences had never experienced this as a formal request, and only employed this practice informally:

“…there is a part where you can give comments… to the editors, and I sometimes do say things like that… “I'm not familiar with this method, I'm not familiar with that”... But they don't ask that question explicitly.”

*Mid-career social science reviewer interview*

Indicating the degree of confidence in aspects of a review offers reviewers a way to feel they have made a meaningful contribution with their review (for example, by focusing on methodological aspects) without needing to comment on areas not covered by their expertise. However, as with collaborative review, if this practice isn’t taken into account by journal editors, it will have no bearing on outcomes for interdisciplinary research papers. If reviewers indicate their confidence using an anonymous channel, such as notes to editors, authors may be unaware of potential expertise gaps within their reviewer panel.

### The role of editors in the peer review of interdisciplinary research

Unsurprisingly, the role of journal editors emerged as a significant theme in both survey and interview data. Editors, associate editors and sub-editors mediate between authors and reviewers. Crucially, throughout the double-anonymised peer review process, editors are aware of author and reviewer identities. They make decisions at the top level, starting with desk-based assessment and assigning reviewers, and they have final discretion over publishing decisions. The degree to which a journal is sympathetic to interdisciplinary research is determined to some degree by the sympathies of individual editors. This issue is addressed by triple-anonymised peer review [5, 6], although uptake of this model is limited.

Reviewers and authors who responded to the survey highlighted the importance of providing proper guidance for reviewers to improve the quality of anonymised peer review. As one comment noted, “As a reviewer I like editorial boards to provide clear guidance on what they are looking for.” The requirement that guidance for reviewers (and authors) be ‘clear’ came up repeatedly in suggestions about enablers. Calls for “clear criteria for evaluating interdisciplinary research”, “clear interdisciplinary expectations from editors/publishers”, “clear journal guidelines that promote interdisciplinary articles” and “clear guidelines to evaluate interdisciplinary integration” suggest that where guidance exists, it is not as comprehensive or comprehensible as reviewers want.

The provision of guidance for reviewers is not a consistent (or even particularly common) practice by journals in general – there is an expectation that reviewers will be able to apply their own experience and expertise. Of the 10 reviewers interviewed for this project, none had received any kind of formal guidance from editors to assist with the evaluation of interdisciplinary research. As a result, the quality of reviews can vary significantly. An early-career social scientist compared two recent experiences:

“…it varies based on the journal. So [journal 1] - oh my goodness, it was so robust… so it was 3 reviewers. One round of review. Each of the reviewers brought a one page review, very detailed, very constructive… It was absolutely great.

I also published a paper in [journal 2] on the other end of the spectrum, absolute shit. They… basically found two reviewers within one week - which wrote like a three or four sentence review… And then we did the feedback and we made some changes and it was published next day. It was absolutely worthless.”

*Early-career social science author interview*

Like finding suitably qualified reviewers, providing guidance on the quality, depth and format of peer-review reports is a responsibility that lies with editors. Consistency of format is increasingly achieved through the use of web-based review platforms, as outlined by a social science editor (emphasis mine):

“…relative to other journals, it's relatively formulaic in that it does ask a set of questions and there's a set of sliders at the end in terms of originality, robustness, contribution and so on. It doesn't say much around interdisciplinarity. There's a kind of implicit assumption that anyone who is happy to identify with… [field], there's a self-selecting sample of people who have some sympathies and understanding of interdisciplinary working.”

*Established social science editor interview*

This “implicit assumption” that reviewers will understand and be sympathetic to interdisciplinary research is in itself likely to be highly contingent on the profile and identity of an individual journal, as in this comment from another associate editor (emphasis mine):

“we're the guidance effectively, because we've got this small group of associate editors, the chief editor assigns them to us… I'll see the report… we write things on positioning, not just on the referee report. So we'll talk about… ‘I think we could write comment for the authors to reposition it in a way that would be more would speak better to the readership of the journal’… that positioning process is institutionalised.”

*Established social science editor interview*

Consistent guidance for reviewers across journals is not only unlikely to be achievable, it is probably not desirable. Not all journals centre interdisciplinary research, although mono-disciplinary journals do occasionally publish interdisciplinary research papers (and would perhaps publish more, if reviewers were properly equipped to assess them). As the quotes above suggest, even within the domain of social science and humanities, guidance practices vary from journal to journal and are based on ‘assumptions’ and informal discussions between editors, rather than formal guidance provided to reviewers. The associate editor’s claim that “we’re [the editorial team] the guidance” gives a glimpse into the otherwise closed box. In the example, the editor acts in a role similar to an additional reviewer, weighing in on how authors of interdisciplinary research publications should ‘position’ their paper for the journal’s readership.

The editors interviewed for this project were candid about the challenges of advocating for interdisciplinary research at their journals. Considering the issues outlined above around aligning reviewer expertise with the disciplinary mix of an interdisciplinary research paper, one editor outlined their approach to ensuring fair process:

“…if an editor feels like a reject is unfair or hasn't been based on an analysis of the whole paper, or has been done by a reviewer who was actually not capable of giving an informed peer review, they can simply invite another reviewer. What you're looking for is at least two endorsements to publish for our journal... So I feel that's really helpful in terms of getting around the usual thing that torpedoes interdisciplinary work.”

*Established social science editor interview*

This approach was also reported from other disciplinary contexts:

“…the peer review process is generally… two reviewers, sometimes three, but they prefer just have two. So if they both kind of broadly align, then that's OK. If they have different opinions then the editor will go and find another one, there's like a judge.”

*Established physical science editor interview*

Editors presented this practice as a way to ensure fair process for interdisciplinary research papers, aimed at mitigating the effects of a reviewer without the necessary expertise – to prevent “the usual thing that torpedoes interdisciplinary work.” In practice, this is an example of the idiosyncrasies of anonymised peer review as a ‘closed box’ and the under-discussed role of editor discretion. While motivated by good intentions in relation to the barriers faced by interdisciplinary research, editor interventions of this kind undermine the process of anonymised peer review, allowing third parties with knowledge of identities to affect decisions. One reviewer reported being on the other side of this practice for a paper that applied social science methods in a physical sciences context:

“I've had an experience with [journal] where… the interviews have not been done very well or… the analysis of the data wasn't done very well, and I pushed them to do more work on that and gave it major revisions. And I think the paper was co-authored by one of the big names in [field] so I think [the editors] just decided… to ignore my comments and to publish.”

Mid-career social science reviewer interview

Cases like this, where one reviewer’s legitimate concerns are over-ruled by an editorial intervention, are clearly in contravention of the principles of anonymised peer review. The ‘closed box’ conceals these practices, which would not be possible in cases where open peer review principles were employed.

## Discussion

These data come with significant limitations which affect the degree to which conclusions can be drawn. The number of survey respondents is low; all participants are *self-identified* interdisciplinary researchers, which limits the possibility of any kind of systematic understanding of interdisciplinary practices (for example, 45% of self-identified interdisciplinary researchers answered ‘no’ when asked if they had direct experience of interdisciplinary co-authoring). As the survey population are all from the same institution there are obvious possibilities for gaps to emerge that mirror gaps in the institution’s makeup, be that disciplinary or demographic.

My position within the organisation also had an influence on the makeup of the survey population. While the survey was circulated across the entire University, responses from social sciences and humanities were disproportionately high compared to the biological and medical sciences and physical sciences and engineering. The impact of this selection is partially mitigated by the interdisciplinary focus; many of the social science and humanities respondents worked closely with colleagues from the other disciplinary bases.

### Open peer review and interdisciplinarity

Could open peer review practices solve the problems that emerge from the peer review of interdisciplinary research? The answer isn’t straightforward. As illustrated by Ross-Hellauer [10], open peer review isn’t one practice but a suite of approaches which are employed in patchwork fashion across a variety of journals. These approaches include ‘open identities’, where reviewers sign their reports; ‘open reports’, where reviewer reports are published alongside the final article; and ‘open participation’, where the opportunity to review is opened up to a wide community, as practiced by journals such as *Atmospheric Chemistry and Physics* [37].

Each of these practices offers some potential advantages and some disadvantages. Requiring *open identities* could reduce what one survey respondent termed “…careless/negligent reviews”, although it would further expose reviewers to accusations of (inter)disciplinary ignorance. *Open reporting* practices could potentially increase the quality of written reviews, and would prevent editorial interventions from passing unnoticed. Critics argue that the risk of recrimination can make reviewers less objective, and open reports would not address issues of disciplinary ‘bias’ or ‘closed-mindedness’ around interdisciplinary research. While reviewees may find this objectionable, reviewers who review from a mono-disciplinary perspective may do so in a perfectly thorough and defensible manner. *Open participation* in the peer review of interdisciplinary research would maximise the pool of (inter)disciplinary expertise available to comment, bypassing the need for reviewers to informally consult with colleagues. In practice open participation approaches have not limited the impact of disciplinary ‘biases’; securing widespread participation is not easy; and quality control on comments requires input from editors [10].

Across the survey and interviews, this study has uncovered three key practices that are already occurring informally in the evaluation of interdisciplinary research papers (and indeed are also affecting the review of mono-disciplinary papers):

- Consultation with expert colleagues;
- Indicating confidence in aspects of a review;
- Editor interventions in assessment processes.

These do not represent inherently ‘good’ or ‘bad’ ways to approach the peer review of research papers. Problems only arise when these practices are obscured – where reviewers rely heavily on input from colleagues and then obscure this fact; where editors overlook a low reviewer confidence score or conceal it from authors; or where editors ignore, over-rule or undermine the recommendations of reviewers without making the decision-making process transparent.

One route forward for the peer review of interdisciplinary research might therefore be to formalise these practices into a coherent framework which employs key principles of open peer review without creating a significant additional burden of work for editors. Drawing on practices of ‘collaborative peer review’ employed by a small number of journals (e.g. the European Molecular Biology Organisation) [38] this framework for ‘interdisciplinary peer review’ could conceptualise the review of interdisciplinary research as an interdisciplinary practice in itself. Hirschauer (cited in 31) argues that a published article is jointly constructed by its authors, editors and peer reviewers, all of whom contribute to shaping the finished piece. The fair assessment of interdisciplinary research could involve making this process of co-creation more visible.

As an exemplar, rather than two (or one, or three) siloed reviewers working on individual reports, a review *team* of two or three could collaborate to produce a single co-authored report. When shared with authors, this would be accompanied by reviewers’ confidence scores on their ability to assess different aspects of the paper (e.g. the topic, the methods employed) and a record of the review team’s discussions. In this way, without adding significant extra effort to the task of assembling a review team, editors can maximise reviewer engagement. Reviewers could consult additional colleagues at their own discretion, with a requirement to disclose this in the report. If reviewer confidence scores happened to indicate a major gap in the review team, editors could use this data to identify where an additional review would be required. It would again be required to disclose this in the report. Although this collective approach would place additional organisational burden on editors, by showing how these decisions were made it would also highlight the role of editors in producing high-quality peer reviews. Additionally, this burden could be mitigated by emerging technologies designed to reduce pressures on editors [39]. Shining a light into the ‘closed box’ may interdisciplinary research papers a stronger chance of fair peer review – and this approach may also be beneficial for mono-disciplinary papers.

## Conclusion

This study sought to add qualitative depth to existing understandings of how interdisciplinary research papers are assessed for quality by reviewers and editors of journals. The review of papers is by nature a closed process and only limited data is available on how decisions are made. By focusing on article review rather than the assessment of research funding proposals, this article makes a contribution at the intersection of interdisciplinary research studies and peer review studies.

A survey and interviews with self-identified interdisciplinary authors, reviewers and editors at a single large research institution revealed that the authors of interdisciplinary papers encounter barriers to publication, some of which overlap with those experienced by authors of mono-disciplinary papers and some more specific to interdisciplinary research. Authors of interdisciplinary research reported negative experiences including rejection based on reviews by reviewers without the necessary expertise to fully assess the work, or by reviewers who evidently did not grasp the purpose or scope of interdisciplinary research. Reviewers, while overall happier with the process of anonymised peer review for interdisciplinary research, reported being assigned papers far outside their normal area of expertise, and in some cases having their recommendations over-ruled by editors without explanation.

The research also uncovered a suite of informal practices undertaken by reviewers and editors intended to redress these barriers, including *collaborative review*, *confidence reporting* and *editor intervention*. While seemingly well-intentioned, these practices are inconsistent with expectations of anonymised peer review [36], and concealing such practices within the so-called ‘black box’ undermines confidence in this kind of review.

Existing open peer review practices, while offering some advantages for the review of interdisciplinary research, fall short of meeting the needs of interdisciplinary author teams. This paper outlines a possible approach to peer review of interdisciplinary research, treating interdisciplinary peer review as a collaborative, interdisciplinary research practice in its own right. While far from solving the many problems that arise in the evaluation of research publications, the proposed model is based on the principle of formalising practices that are already happening.

## Data Availability

De-identified data may be obtained on request from the author.
